# The Supraclavicular Flap for Reconstruction of Post-Burn Mentosternal Contractures

**DOI:** 10.5812/ircmj.1600

**Published:** 2013-04-05

**Authors:** Shahriar Loghmani, Mohammad Eidy, Mahdi Mohammadzadeh, Alireza Loghmani, Fahimeh Raigan

**Affiliations:** 1 Isfahan University of Medical Sciences, Isfahan, IR Iran; 2 Trauma Research Center, Kashan University of Medical Sciences, Kashan, IR Iran

**Keywords:** Surgical Flap, Contracture

## Abstract

**Background:**

The thin and pliable skin of the neck is a region with multidirectional activity, and postburn scar contractures tend to form there easily. The supraclavicular flap is used to correct neck scar contractures. Its main vascular supply is the supraclavicular artery, and it can be harvested as either a skin pedicle flap or an island flap (vascular pedicle flap).

**Objective:**

In this article, a total of 41 flaps are studied retrospectively and their efficacy in reconstruction of post-burn neck scar contractures is discussed. Also donor-site morbidity, patient satisfaction, and complications were evaluated.

**Patients and Methods:**

Between November of 2004 and January of 2009, 41 supraclavicular flaps were used for reconstructions in 32 patients at the authors’ hospital. Twenty-four of these flaps were skin pedicle flaps, and 17 were island flaps. The range of flap size was 18 ± 6 cm in length, and 9 ± 3 cm in width. Pre-expansion was performed in 14 flaps. Primary closure of donor site was performed in 35 flaps.

**Results:**

Thirty-seven of the 41 flaps survived completely, but there were three cases of distal necrosis (10-30%), and one case of complete flap necrosis. Twenty-nine of the 32 patients were satisfied with both the functional and aesthetic results.

**Conclusions:**

Scarring of the neck produces problems with function, and appearance. In our view, the supraclavicular flap, a thin flap of good texture, is an excellent and highly reliable flap for covering defects of the anterior neck. This flap is easy to harvest, with good functional and aesthetic results.

## 1. Background

Burn scar contractures involving the anterior cervical neck present a unique set of problems compared with the rest of body. The thin and pliable skin of the neck is a region with multidirectional activity, and postoperative scar contractures tend to form there easily. Scars in this area may lead not only to restriction in the range of motion of the neck but also to abnormal function of the lower face, such as lip incompetence, and reduced facial expression. In the developing child, scar contractures in the cervical region may lead to growth disturbances of the mandible, and the spine. The challenge lies in restoring the form and function of this region.

Many methods have been applied for neck reconstruction, with perforator flaps, “superthin flaps,” (1, 2), and expanded flaps all in common use. Each has its own advantages, and disadvantages. The flap based on the vascular net of the cervical artery and its branch, the supraclavicular artery, is not new, and has a long history of controversy as a surgical method of choice. Skin expansion in the donor region not only allowed coverage of the larger unit of the anterior neck but also modified the morphologic characteristics of the transferred flap through capsule formation, and fatty tissue atrophy, which was beneficial for obtaining an optimal neck reconstruction. Since 2004, we started using the supraclavicular flap for reconstruction of head and neck defects, and for reconstruction of the thoracic wall. We used this flap as skin pedicle, island flap and pre-expanded. In this article, a total of 41 flaps were studied retrospectively, and their efficacy in reconstruction of postburn neck scar contractures is discussed. Also donor-site morbidity, patient satisfaction, and complications were evaluated.

## 2. Objectives

In this article, a total of 41 flaps are studied retrospectively and their efficacy in reconstruction of post-burn neck scar contractures is discussed. Also donor-site morbidity, patient satisfaction, and complications were evaluated.

## 3. Patients and Methods

### 3.1. Flap Design and Elevation

**Figure 1.**
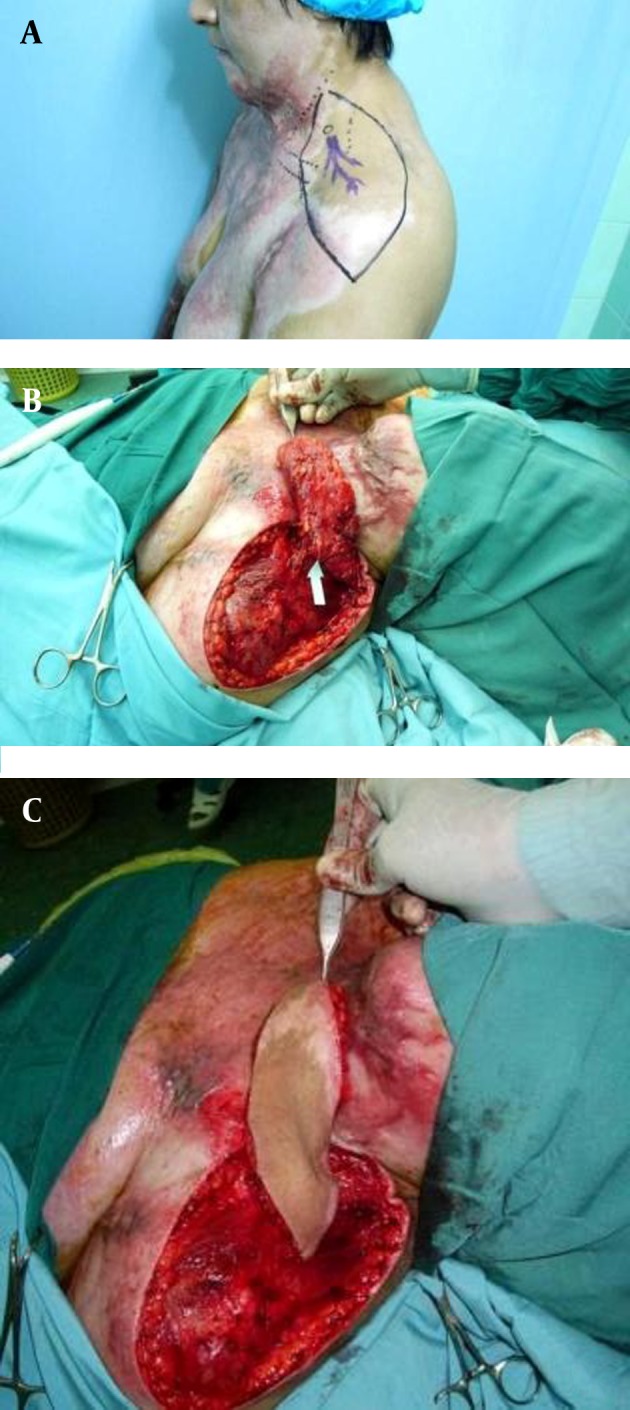
Flap Design and Preoperative Outline of the Flap Pedicle byMeans of the Doppler Probe (1a). Intraoperative View of the Harvested Flap (measuring 23 × 10 cm) After Isolating the Vascular Pedicle (arrow) (1b). The Island Flap Was Elevated, and Rotated 180 Degrees to Cover the Defect (1c).

Flap size is determined according to the recipient site, and the flap is designed on the patient’s shoulder. We generally prefer fusiform designs, as they allow the donor site to be closed primarily (Figure 1a), the choice of island flap or skin. Pedicle flap is made according to the condition of the recipient site. It is possible to harvest flaps up to 11 cm in width, and 21 cm in length, allowing their anterior edge to reach the inferior border of the clavicle, the posterior edge to reach the upper area of the trapezius muscle, and the distal edge to reach the upper arm. Doppler flowmetry is useful for identifying the supraclavicular artery. During the operation, recipient site scars were debrided to the depth of the deep fascia to release the contractures completely. The harvested flap includes skin, subcutaneous tissue, and the fascia of the deltoid muscle. In the medial part of the flap, the supraclavicular artery, which arises from the transverse cervical artery in most cases, can be identified (Figure 1b). In some cases, we refine the transverse cervical artery, and its bifurcation from the supraclavicular artery to enable harvesting an island flap with a longer pedicle. When this is performed, the pivot point of the flap becomes the origin of the transverse cervical artery arising from the subclavicular artery. Island flaps can be rotated up to 180 degrees, and cover a much longer area (approximately 4 to 5 cm) than skin pedicled flaps (Figure 1c). The donor site can be closed primarily when the flap width is smaller than 10 cm. When the donor sites are over 10 cm in width, split-thickness skin grafts or local flaps harvested from the dorsal region are necessary.

### 3.2. Patients

Between November 2004 and January 2009, we used 41 supraclavicular flaps in 32 patients (22 female patients, and 10 male patients) (Table 1). All cases underwent the procedure for postburn neck scar contractures at Emam Musa Kazem Burn Hospital. The mean age at first examination was 24.62 years, and the age range was 12 to 46 years. Twenty-four of these flaps were skin pedicle flaps, and 17 were island flaps; three of them were transferred through skin tunnels to reduce continuous scar formation between the donor, and recipient sites. Pre-expansion was performed in 14 cases (Table 1). Tissue expansion was performed only in cases of unilateral supraclavicular flaps. Delay was not used in our cases. Skin grafting of donor site was performed in 6 cases; primary closure was performed in the others. The range of flap size was 18 ± 6 cm in length, and 9 ± 3 cm in width. Fourteen patients had previous skin grating of anterior neck.

**Table 1.** Patient Information

Case	Age, y	Sex	Donor Site Closure	Flap Size, cm	Pedicle	Pre-expansion	Complications
**1**	12	M	Primary	12×6	RT	NO	NO
**2**	17	F	Primary	17×10	LT	YES	NO
**3**	46	F	STSG	24×12	RT	NO	100% necrosis
			Primary	17×10	LT	NO	NO
**4**	24	F	Primary	18×9 18×8	RT	NO	NO
			Primary		LT	NO	NO
**5**	23	F	Primary	17×10	LT	NO	NO
**6**	21	F	Primary	18×10	RT	YES	hematoma
**7**	33	M	Primary	18×8	RT	YES	NO
**8**	22	F	Primary	17×10	LT	NO	NO
**9**	14	M	Primary	17×8	RT	NO	NO
**10**	38	F	Primary	17×10	RT	NO	NO
			Primary	16×8	LT		
**11**	41	F	Primary	19×10	LT	YES	30% necrosis
**12**	42	F	Primary	18×9	LT	YES	NO
**13**	15	M	Primary	17×8	LT	YES	NO
**14**	21	F	STSG	23×12	RT	NO	10% necrosis
**15**	23	F	Primary	18×10	LT	YES	NO
**16**	20	F	Primary	16×8	RT	NO	NO
			Primary	20×10	LT	NO	20% necrosis
**17**	18	M	Primary	17×8	RT	NO	NO
			STSG	20×11	LT	NO	NO
**18**	25	M	Primary	18×9	LT	NO	NO
**19**	16	F	Primary	18×10	RT	NO	NO
					LT	NO	NO
**20**	37	F	Primary	18×10	LT	YES	NO
**21**	16	M	Primary	17×8	RT	YES	NO
**22**	30	F	Primary	18×10	RT	NO	hematoma
**23**	41	F	STSG	21×11	RT	NO	NO
			Primary	17×8	LT	NO	NO
**24**	15	M	Primary	18×8	LT	NO	NO
**25**	26	F	STSG	20×12	LT	YES	NO
**26**	21	F	Primary	18×10	LT	YES	NO
**27**	28	F	Primary	18×9	LT	YES	NO
**28**	17	F	Primary	17×8	RT	NO	NO
**29**	21	M	Primary	18×8	RT	NO	NO
				21×9	LT	NO	NO
**30**	19	M	Primary	17×8	LT	YES	NO
**31**	29	F	Primary	21×11	RT	NO	NO
			STSG	16×9	LT	NO	NO
**32**	17	F	Primary	18×9	LT	YES	NO

### 3.3. Case 1

A 34-year-old woman sustained severe flame burns on the neck and chest (Figure. 2). Emergency split-thickness skin grafts were applied, but neck scar contractures developed postoperatively. The right shoulder had not sustained burns, so neck reconstruction using a unilateral supraclavicular island flap measuring 20 x 12 cm was designed. The flap was elevated as an island flap, and transferred to cover the defect after removal of the scar. It survived completely, and the functional and aesthetic results were good, although a scar band remained on the left neck. We are planning to remove this in the near future. The donor site was closed with a split-thickness skin graft.

### 3.4. Case 2

A 28-year-old woman sustained flame burns, and scar contractures developed on his neck one year after the application of skin grafts (Figure 3). A unilateral supraclavicular island flap (18 x 9 cm), and ipsilateral small rotation skin flap were designed for reconstruction. After removal of the scar, the island flap was elevated, and rotated 180 degrees to cover the defect. The two flaps survived completely and there were no complications, the donor site has been closed primarily. The texture and color match were good, and full functioning of the neck was recovered.

### 3.5. Case 3

A 25-year-old woman sustained severe flame burns on the neck and chest (Figure 4). Emergency split-thickness skin grafts were applied, but neck scar contractures developed postoperatively. Both shoulders had not sustained burns, so neck reconstruction using a bilateral supraclavicular flaps measuring 17 x 9 cm, and 15 x 8 cm was designed. The flaps were elevated as skin pedicle flaps, and transferred to cover the defect after removal of the scar. They survived completely, and the functional and aesthetic results were good, although a scar band remained on the left neck. We are planning to remove this in the near future. The donor sites have been closed primarily.

## 4. Results

Twenty-nine of the 32 patients were satisfied with both the functional and aesthetic results. Thirty-seven of the 41 flaps survived completely, but there were three cases of distal necrosis (10-30%), and one case of complete flap necrosis. In cases of distal necrosis, raw surfaces epithelialized naturally in two of them; and the third required a split full-thickness skin graft after debridement of the necrosis tissue. In the patient with complete flap necrosis reconstruction was performed with a skin graft. The causes of distal (or complete) flap necrosis were considered to be injury to the supraclavicular artery during operation, kinking of vascular pedicle, tight dressing or tension. Closed suction drain was inserted below the flap in all cases. In the postoperative period, there were two cases of hematoma which treated successfully with drainage, irrigation, and hemostasis. No case of infection was observed. Some patients may need revisions and further interventions as well as laser touch ups for restoration of near normal neck contour and correction of dog ears. The second revisional operation is needed in 24 cases. This stage was performed 3-6 weeks after the primary flap transfer.

## 5. Discussion

The cervical region is functionally and anatomically designed to achieve a maximum range in three-dimensional motion. Furthermore, the cervical area, as does the facial region, functions as a medium to interact with human society. Mentosternal contractures are well-known complications after burns, scald injuries, and injuries with acid or lye. In addition, the neck is a sensitive region, not only from the functional but also from the aesthetic point of view, and in burn patients, especially disfigurations such as ectropion of the eyelids, and lips are liable to occur. Many methods have been applied for neck reconstruction. The supraclavicular flap offers high reliability in addition to its thinness. Skin from the supraclavicular area is considered a good match for reconstruction of the face, and neck regions. With expanded supraclavicular skin flap, we can increase its size and reach. This method is easier to perform compared with the supraclavicular island flap, and is particularly attractive in patients who are at risk for poor or delayed healing, such as smokers or patients with complex medical histories in whom longer, and more complex procedures may be associated with increased risk (b). It was first described by Kazanjian and Converse as “in charretera” or acromial flap (3). Today’s supraclavicular flap represents the evolution of several other flaps such as the Demergasso flap (4) which was defined by Mathes and Nahai as a fasciocutaneous extension of the lateral superior trapezius flap on the territory of the lateral aspect of the arm (5-7) Lamberty and Cormack defined the flap as a laterally extended cervicohumeral flap, and described two pivot points (8, 9) The flap, widely is used in the restoration of the cervicofacial region by Mathes and Vasconez (10). In the 1970s, was criticized by Blevins and Luce (11) because of the high incidence of necrosis of the distal part of the flap when elevated in its maximal extension. The "supraclavicular flap" was reported for the first time by Lamberty in 1979 as a thin and pliable fasciocutaneous flap (12). In 1983, Lamberty and Cormack (13) published an article about the vascular anatomy of the flap to demonstrate its reliability, although the use of the flap progressively decreased, and no more mention of it was made in the literature after that date. The transverse cervical artery courses posteriorly across the base of the posterior triangle of the neck, behind or posterior to the inferior belly of the omohyoid muscle. The supraclavicular artery is a perforator which arises from the transverse cervical artery in 93 percent of cases or from the suprascapular artery in 7 percent (13). It divides into one or two arteries before reaching the deep fascia of the deltoid muscle in the third medial clavicle. These one or two arteries then run toward the acromioclavicular joint, where they further divide into small branches to reach the superior part of the deltoid muscle. At this level, they become interfused with the cutaneous branches of the posterior circumflex humeral artery. The supraclavicular artery is also interfused with the vascular network of the musculocutaneous perforator of the trapezius muscle in the dorsal region, and with the vascular network of the cutaneous branches of the thoracoacromial artery in the anterior chest (12, 13). In 1997, Pallua et al. (14) published a modification of the flap, defining it as a “supraclavicular artery island flap.” The flap first used for releasing postburn mentosternal contractures was used for several other indications. In 2000, Pallua and Noah (15) again published a detailed anatomical study about the shoulder cap, exactly describing the vascularity of the supraclavicular island flap, and the possibility of tunneling it to improve the mobility of the flap, and to reduce the donor-site scar. After the first article by Pallua et al. the flap reacquired its dignity as a reliable, and anatomically based technique which can be successfully used in particular reconstructive problems. Then in 2005 Pallua et al. presented a novel modification of a previously published method as pre-expanded ultrathin supraclavicular flaps for face reconstruction. Advantages of his method were low donor site morbidity, and no need for microsurgery (16). In 2005, Di Benedetto et al., introduced a modification of the supraclavicular island flap, harvesting it as a supraclavicular flap with a facial pedicle, thus reducing the risk of flap necrosis (17). In 2005, Heitland et al. introduced the single and double-folded supraclavicular island flap in the treatment of large facial defects in noma patients (18). In 2007, Margulis et al. introduced the prefabricated expanded supraclavicular flap carried on the thoracoacromial pedicle for providing ample tissue for local transfer. This flap was successfully used for reconstruction of the anterior neck, with good functional, and aesthetic results (19). We have achieved good results with it in 37 of 41 flaps (90.24 percent), which nearly match the rate reported by Pallua and Machens (87.5 percent) (14). Also Vinh et al. (20) reported good results with supraclavicular flap in 86.7 percent of cases of neck contractures. In our experience, Advantages of this flap were low donor site morbidity, highly reliability, ease of dissection, and no need for microsurgery. We have also found that the supraclavicular flap can be safely elevated provided that it is within 20 x10 cm. With larger defects, the bilateral supraclavicular flap is an alternative choice. Sometimes for achieving larger flaps we select expanded supraclavicular flaps. Scarring of the neck produces problems with function and appearance. In our view, the supraclavicular flap, a thin flap of good texture, is an excellent and highly reliable flap for covering defects of the anterior neck. This flap is easy to harvest, with good functional and aesthetic results. Because of its simplicity and reliability actually reducing the indication for free flaps to the neck in cases of large full-size defects. If a supraclavicular flap wider than 10 cm is harvested, skin grafts or small cutaneous flaps should be used for donor-site closure. If the skin defect of neck is larger than 10x20 cm pre-expansion of flap or bilateral supraclavicular flap are alternative choices.
